# Dual specificity phosphatase 6 deficiency is associated with impaired systemic glucose tolerance and reversible weight retardation in mice

**DOI:** 10.1371/journal.pone.0183488

**Published:** 2017-09-05

**Authors:** Katrin Pfuhlmann, Paul T. Pfluger, Sonja C. Schriever, Timo D. Müller, Matthias H. Tschöp, Kerstin Stemmer

**Affiliations:** 1 Research Unit NeuroBiology of Diabetes, Helmholtz Diabetes Center, Helmholtz Zentrum München, Neuherberg, Germany; 2 Institute for Diabetes and Obesity, Helmholtz Diabetes Center, Helmholtz Zentrum München, Neuherberg, Germany; 3 German Center for Diabetes Research (DZD), Neuherberg, Germany; 4 Division of Metabolic Diseases, Technische Universität München, Munich, Germany; Universidade de Santiago de Compostela, SPAIN

## Abstract

Here, we aimed to investigate the potential role of DUSP6, a dual specificity phosphatase, that specifically inactivates extracellular signal-regulated kinase (ERK), for the regulation of body weight and glucose homeostasis. We further assessed whether metabolic challenges affect *Dusp6* expression in selected brain areas or white adipose tissue. Hypothalamic *Dusp6* mRNA levels remained unchanged in chow-fed lean vs. high fat diet (HFD) fed obese C57Bl/6J mice, and in C57Bl/6J mice undergoing prolonged fasting or refeeding with fat free diet (FFD) or HFD. Similarly, *Dusp6* expression levels were unchanged in selected brain regions of Lep^ob^ mice treated with 1 mg/kg of leptin for 6 days, compared to pair-fed or saline-treated Lep^ob^ controls. Dusp6 expression levels remained unaltered *in vitro* in primary adipocytes undergoing differentiation, but were increased in eWAT of HFD-fed obese C57Bl/6J mice, compared to chow-fed lean controls. Global chow-fed DUSP6 KO mice displayed reduced body weight and lean mass and slightly increased fat mass at a young age, which is indicative for early-age weight retardation. Subsequent exposure to HFD led to a significant increase in lean mass and body weight in DUSP6 deficient mice, compared to WT controls. Nevertheless, after 26 weeks of high-fat diet exposure, we observed comparable body weight, fat and lean mass in DUSP6 WT and KO mice, suggesting overall normal susceptibility to develop obesity. In line with the increased weight gain to compensate for early-age weight retardation, HFD-fed DUSP6 KO displayed increased expression levels of anabolic genes involved in lipid and cholesterol metabolism in the epididymal white adipose tissue (eWAT), compared to WT controls. Glucose tolerance was perturbed in both chow-fed lean or HFD-fed obese DUSP6 KO, compared to their respective WT controls. Overall, our data indicate that DUSP6 deficiency has limited impact on the regulation of energy metabolism, but impairs systemic glucose tolerance. Our data are in conflict to earlier reports that propose protection from diet-induced obesity and glucose intolerance in DUSP6 deficient mice. Reasons for the discrepancies remain elusive, but may entail differential genetic backgrounds, environmental factors such as the type and source of HFD, or alterations in the gut microbiome between facilities.

## Introduction

In the late 20th century and new millennium, obesity has evolved from an isolated problem of the rich and wealthy into a widespread global epidemic that spans all social classes. Understanding the underlying molecular mechanisms by which an organism becomes obese is a prerequisite for successful weight loss therapies. Obesity is characterized by an increase in adipose tissue mass that includes increased fat cell size and fat cell number. The latter is characterized by differentiation of fibroblast-like pre-adipocytes into mature adipocytes, and governed via tightly controlled activation and inactivation of mitogen activated protein kinases (MAPKs). These highly conserved serine/threonine kinases control the interplay between cell differentiation, cell proliferation and cell death in multiple cell types [1].

Extracellular signal-regulated kinase (ERK) stands out as important positive [2,3][4,5] and negative [6] regulator of the adipocyte differentiation process. Mice with global (germline) ablation of ERK1 were shown to have decreased adiposity when fed high-fat diet (HFD), which was attributed to impaired adipogenesis as well as increased thermogenesis [4]. The plurality of ERK action towards multiple physiological processes, including adipocyte differentiation, demands tight control of spatial and temporal ERK activity in relation to the physiological state of the cell. Canonical RAF/MEK/ERK signaling, is activated by intracellular mitogenic and stress stimuli as well as extracellular hormones such as leptin or ghrelin [7]. Fine-tuning of canonical ERK signaling is achieved by scaffolding proteins such as kinase suppressor of RAS 1 (KSR1) [8,9] or KSR2 [10], which support the interaction of ERK with RAF/MEK or the phosphatase calcineurin, respectively, to localize active ERK to specific membrane microdomains and substrates [11]. The duration and intensity of ERK activation is further modulated by dual-specificity phosphatases (DUSPs), which inactivate ERK by dephosphorylating its serine/threonine and tyrosine residues [12–14].

At current, 38 DUSP family members are known, and 11 use MAP kinases as substrate; of those, only DUSP6 displays high selectivity for ERK1/2, the others are promiscuity towards JNK, p38 or ERK [15]. *Dusp6* is expressed in multiple tissues, with highest expression in the brain [16], adipose tissue [17], heart and pancreas [15] and has been implicated in multiple physiological processes ranging from brain development [18] and heart function [19] to tumorigenesis [20–22].

Adult mice with DUSP6 deficiency displayed increased basal ERK activity states in multiple organs such as heart, spleen, kidney and brain [19]. DUSP6 deficiency was further linked with impaired adipocyte differentiation *in vitro*, and with decreased propensity for diet-induced obesity and pernicious sequelae in mice chronically exposed to HFD [23]. In hepatocytes, DUSP6 was shown to mediate forkhead box protein O1 (FOXO1) dephosphorylation and nuclear translocation, which allowed an increased activation of gluconeogenic gene programs and glucose output [17,24]. Accordingly, diminished hepatic *Dusp6* expression was associated with improved glucose homeostasis in both diet-induced obese (DIO) and leptin deficient (Lep^ob^) mice [25].

Our study aimed to assess a potential weight- and glucoregulatory role of DUSP6 in mice. We moreover aimed to assess whether *Dusp6* gene expression in adipose tissue and the hypothalamus is directly affected by dietary and hormonal challenges. In contrast to previous studies, DUSP6 deficiency had only little impact on the regulation of energy metabolism or lipid homeostasis. Moreover, rather than improving glucose tolerance, 16 weeks of HFD feeding resulted in temporarily increased glucose excursions after a glucose bolus.

## Methods

### Mouse husbandry

Global DUSP6 wild type (WT) and knockout (KO) mice of mixed C57Bl/6J and 129 background were kindly provided by Prof. Jeffery Molkentin, Children’s Hospital Cincinnati [19]. Study cohort mice were bred true from WT and KO mice, and housed in positive individual ventilation cages in dedicated animal housing rooms with a 12-h light, 12-h dark cycle (6am-6pm) at 22°C with free access to water and standard chow diet (Harlan Teklad LM-485) or high fat diet (HFD; Research Diets D12331 Surwit Diet, 58% of calories from fat, New Brunswick, NJ, USA). Initially, all mice were housed in groups of 2 to 4. By the end of the diet exposure, 6 WT mice were housed 2 per cage, 2 WT mice were single housed, 6 KO mice were housed 2 per cage and 3 KO mice 3 per cage due to fighting. Food intake was assessed in mice exposed to HFD from week 3 to 5, with 3 WT mice being temporarily single housed to increase statistical power. One cage of WT mice was excluded from the analysis due to spilling of the food.

For the analysis of Dusp6 expression in lean vs. diet induced obese (DIO) mice, C57Bl/6J mice (Jackson Laboratory, Bar Harbor, ME, USA) with an age of 8 to 10 weeks were either maintained on chow or switched to HFD for 6 months. The impact of leptin on the central nervous system (CNS) *Dusp6* gene expression was assessed using adult chow-fed leptin-deficient (Lep^ob^) mice (71.12 ± 0.71 g) that once daily received subcutaneous injections of either human recombinant leptin (1 mg/kg; R&D Systems) or vehicle (PBS). An additional group of vehicle-treated Lep^ob^ mice was food restricted to receive daily only the amount of food eaten by the leptin-treated mice (pair-fed to leptin). To assess the impact of fasting and re-feeding on *Dusp6* gene expression, male chow-fed C57BL/6J mice were subjected to 12, 24 or 36 h of fasting as well as refeeding for 6 h with fat-free diet (FFD; 0% kcal from fat, D04112303 Research Diets) or HFD [26]. Mice were sacrificed by asphyxiation with CO_2_. All studies were approved by and performed according to the guidelines of the Institutional Animal Care and Use Committee of the University of Cincinnati, USA.

### Glucose tolerance tests

Following 16 weeks of HFD feeding, DUSP6 WT and KO mice were fasted for 6 h, and subsequently subjected to an intraperitoneal injection of 2 g glucose per kg body weight (20% wt/vol D-glucose in 0.9% wt/vol saline). Tail blood glucose levels (mg dl-1) were measured with a handheld glucometer (TheraSense Freestyle) before (0 min) and at 15, 30, 60 and 120 min after injection. Area under the curve values were calculated by using the statistical software GraphPad Prism.

### Primary adipocyte cell culture and differentiation

Primary murine pre-adipocytes were obtained from subcutaneous white adipose tissue of four 6- to 8-weeks old male C57BL/6J mice. Fat pads were kept separate during the isolation procedure to obtain four biological replicates. Fat pads were dissected and minced in phosphate-buffered saline (PBS) using spring scissors. Minced tissues were transferred to digestion buffer containing collagenase IV (Life Technologies; 1 mg/ml), dispase II (Sigma Aldrich; 3 U/ml) and CaCl_2_ (Sigma Aldrich; 0.01 mM) in PBS. Tissues were digested while shaking at 37°C for 50 min. Homogenates were filtered through a 100 μm strainer. The strainer was cleared with growth media consisting of DMEM/F12 + glutaMAX^TM^ (Thermo Fisher Scientific, #31331028) with 10% FBS and 1% penicillin streptomycin, and cells were centrifuged at 500 x g for 10 min at room temperature. After removal of the supernatant the cells were re-suspended in growth media and again filtered through a 70 μm strainer. Cells were re-suspended in growth media, centrifuged at 500 x g for 10 min at room temperature, re-suspended in growth media, seeded in cell culture plates, and maintained in an incubator with 10% CO_2_. The day after extraction and then every other day the medium was changed. Before cells reached confluence, living cells were counted and 0.15 x 10^5^ cells were seeded per well in 12-well plates. One day after cells reached confluence, differentiation was induced with growth media supplemented with dexamethasone (Sigma Aldrich; 1 μM), IBMX (3-isobutyl-1-methylxanthine; Biomol; 0.5 μM), rosiglitazone (Santa Cruz; 1 μM) and insulin (Sigma Aldrich; 5 μg/ml). Two days later and every other day until the end of the study, media were changed to growth media supplemented with insulin (5 μg/ml). Plates were frozen before induction of differentiation (day 0) and 2, 4 and 8 days after the start of the differentiation process.

### Oil Red O staining

Oil Red O (0.35% Oil Red O in Isopropanol, Sigma-Aldrich, O-0625) was mixed with dH_2_O (v:v 6:4). Adipocytes were fixed with 4% paraformaldehyde and washed three times with PBS prior to staining. Cells were dried and the Oil Red O solution added for 10 min. Subsequently, cells were washed four times with dH_2_O and dried. Pictures were taken with an EVOS XL Core Cell Imaging System (Thermo Fisher Scientific Inc., Waltham, Massachusetts MA, USA).

### Real-time gene expression

RNA was isolated from brain areas, eWAT and primary adipocytes using a commercially available kit (RNeasy, Qiagen, Hilden, Germany). For qPCR analyses of tissues, equal amounts of RNA were transcribed to cDNA using Superscript III (Invitrogen, Darmstadt, Germany). For qPCR analyses of primary adipocytes, 0.5 μg RNA were transcribed to cDNA using the QuantiTect Reverse Transcription kit (Qiagen, Hilden, Germany). Gene expression levels were assessed on a 7600HT TaqMan Fast Real-Time PCR System (Applied Biosystems, Carlsbad, CA, USA) by using either TaqMan probes (*Dusp6*: Mm00518185_m1, *Hprt*: Mm01545399_m1, Thermo Fischer Scientific, Inc., Rockford, IL USA) and the respective TaqMan mastermixes (Applied Biosystem, Carlsbad, CA, USA), or by using custom-to-order TaqMan low-density Arrays (Applied Biosystems, Carlsbad, CA, USA) as previously described [27].

### Statistical analyses

Statistical analyses were performed by using GraphPad Prism (GraphPad Software, Inc. La Jolla, CA, USA). Two groups were compared by two-tailed unpaired Student's t test, three or more groups by one- or two-way ANOVA followed by Bonferroni post hoc tests, as indicated. P values lower than 0.05 were considered statistically significant. Results represent means ± SEM.

## Results

### Central *Dusp6* expression is not affected by leptin, diet-induced obesity or prolonged fasting

*Dusp6* is highly expressed in the brain of developing as well as adult mice [16]. A comparison of 30 mouse strains further revealed an association between allelic *Dusp6* variant I62M (rs13480726) and forebrain weight and structure [18]. Here, we aimed to delineate the distribution of *Dusp6* mRNA in selected brain areas of leptin-deficient Lep^ob^ mice, and assess the impact of leptin on *Dusp6* expression. We furthermore aimed to assess in C57Bl/6J mice whether hypothalamic *Dusp6* expression is regulated by diet-induced obesity or prolonged fasting and refeeding.

**Fig 1:**
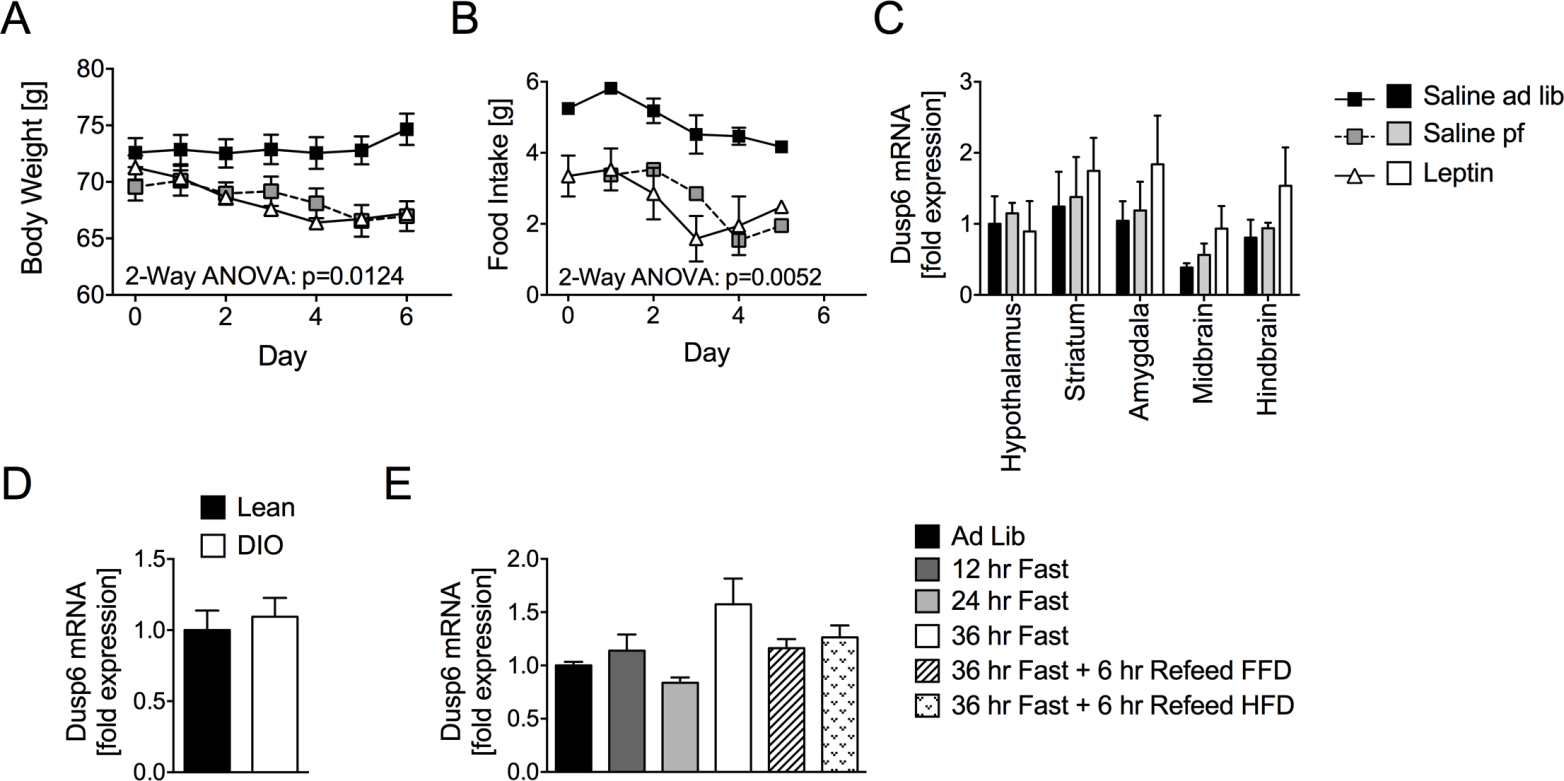
Central *Dusp6* expression is not affected by changes in body composition or nutrition. Decreased body weight (A) and food intake (B) but unchanged levels of *Dusp6* mRNA in brain areas (C) of chow-fed Lep^ob^ mice treated daily for 6 days with 1 mg/kg of leptin (Leptin) and chow-fed Lep^ob^ mice treated with saline but pair-fed (Saline pf) to the Leptin group, compared to ad libitum fed saline controls (Saline *ad lib*) (n = 5–6 mice; food intake: n = 3 cages). *Dusp6* mRNA levels were also assessed in (D) chow-fed lean vs. HFD-fed C57B/6J mice (n = 6–8), and in (E) chow-fed lean C57Bl/6J undergoing fasting or refeeding with fat free or high-fat diet (FFD/HFD; n = 6–8). Means ± SEM.

First, we assessed the impact of leptin on CNS *Dusp6* gene expression in adult ad libitum (ad lib) chow-fed leptin-deficient (Lep^ob^) mice (71.12 ± 0.71g) treated either with human recombinant leptin (leptin) or vehicle saline (saline ad lib). An additional group of saline-treated Lep^ob^ mice was pair-fed to the lower food consumption of the leptin-treated mice (saline pf). Six days of subcutaneous treatment with 1 mg/kg leptin significantly decreased body weight and food intake, as expected (Fig 1A and 1B). Saline pf mice displayed a similar decrease in body weight. Subsequent qPCR analyses *in hypothalami of* saline ad lib mice displayed similar *Dusp6* expression in the hypothalamus, striatum, amygdala, midbrain and hindbrain (Fig 1C). Moreover, leptin treatment had no effect on *Dusp6* expression compared to Saline *ad lib* or Saline pf mice (Fig 1C). In adult C57Bl/6J mice exposed to chow-diet or HFD for 6 months, we found similar hypothalamic *Dusp6* levels (Fig 1D).

We next aimed to assess the impact of a chronically negative energy balance on hypothalamic *Dusp6* expression levels, and subjected 8-week-old chow-fed C57Bl/6J mice to 12, 24 or 36 h of fasting. Moreover, by applying refeeding with fat free diet (FFD) vs. HFD, we aimed to delineate the impact of circulating free fatty acids and other lipid species, which are known to surge after both prolonged fasting and HFD exposure, from the impact of other macronutrients such as protein or carbohydrates. Quantitative PCR analyses neither showed alterations of hypothalamic *Dusp6* mRNA levels by fasting nor by FFD or HFD feeding, which indicates that both circulating lipids and a chronically negative energy balance have no impact on hypothalamic *Dusp6* expression (Fig 1E).

### Diet-induced obesity induces *Dusp6* mRNA levels in white adipose tissue

**Fig 2:**
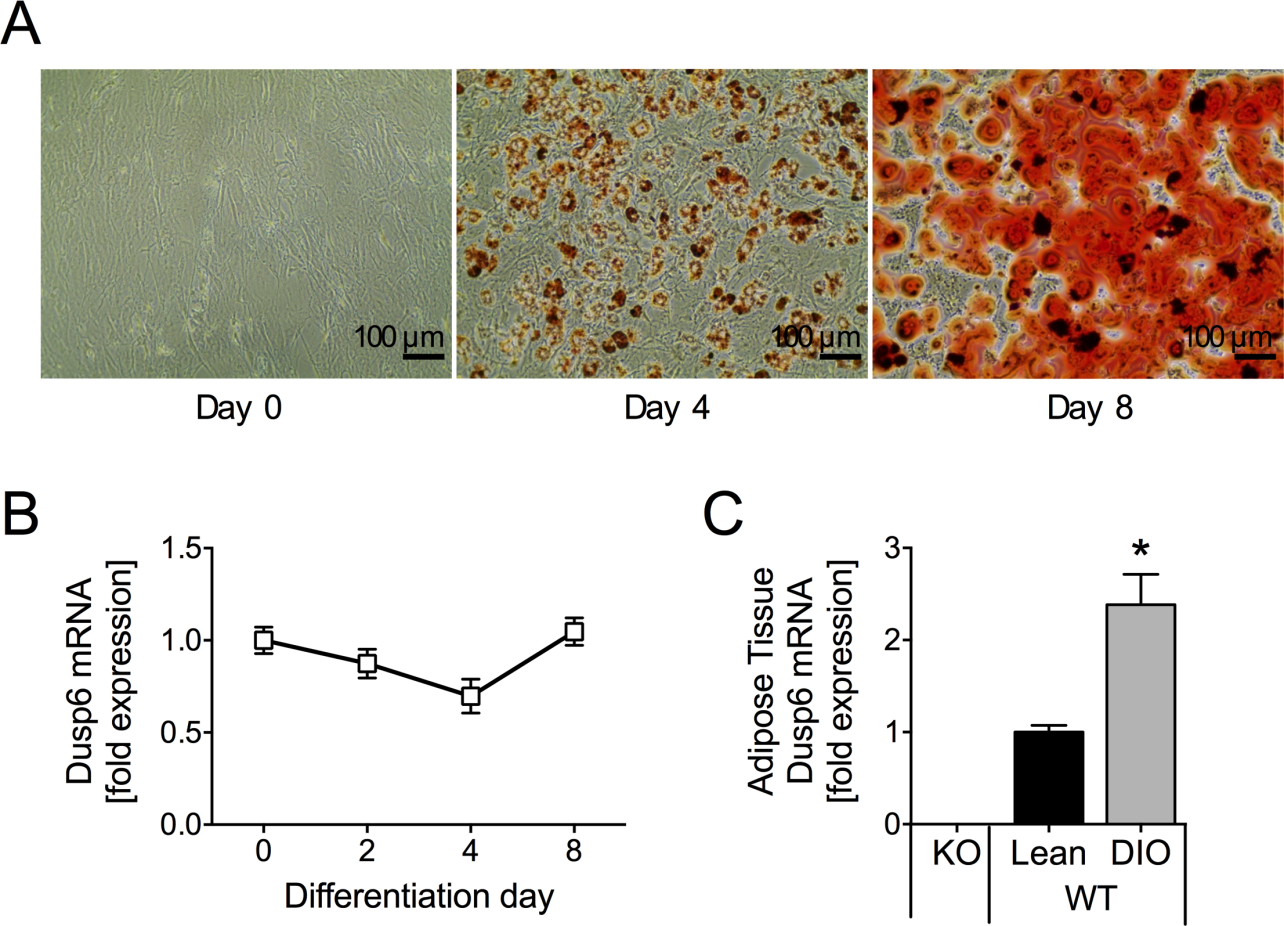
Diet-induced obesity increases *Dusp6* expression in eWAT. Primary pre-adipocytes isolated from inguinal WAT of C57Bl/6J mice undergoing differentiation to mature adipocytes displayed increased triglyceride content (A; representative images Oil Red O staining), but unchanged *Dusp6* mRNA levels (B; two independent wells from two cell preparations). *Dusp6* mRNA was absent in epididymal WAT of HFD-fed DUSP6 KO mice, and increased in HFD-fed obese compared to chow-fed lean C57Bl/6J mice (C; n = 3–8). Means ± SEM, *p<0.05.

We next examined whether Dusp6 is expressed *in vitro* in primary adipocytes from inguinal white adipose tissue (iWAT), and altered during differentiation from pre-adipocytes into adipocytes. Oil Red O staining revealed a profound increase in triglycerides during differentiation (Fig 2A), but unaltered *Dusp6* expression (Fig 2B). *In vivo*, we assessed whether diet-induced obesity can alter *Dusp6* expression in epididymal white adipose tissue (eWAT). *Dusp6* mRNA levels were absent from eWAT of KO mice, as expected. In mice exposed to HFD for 6 months we observed a significant increase in *Dusp6* expression in eWAT, compared to chow-fed lean controls (Fig 2C).

### Decreased body weight and altered body composition in young Dusp6-deficient mice

**Fig 3:**
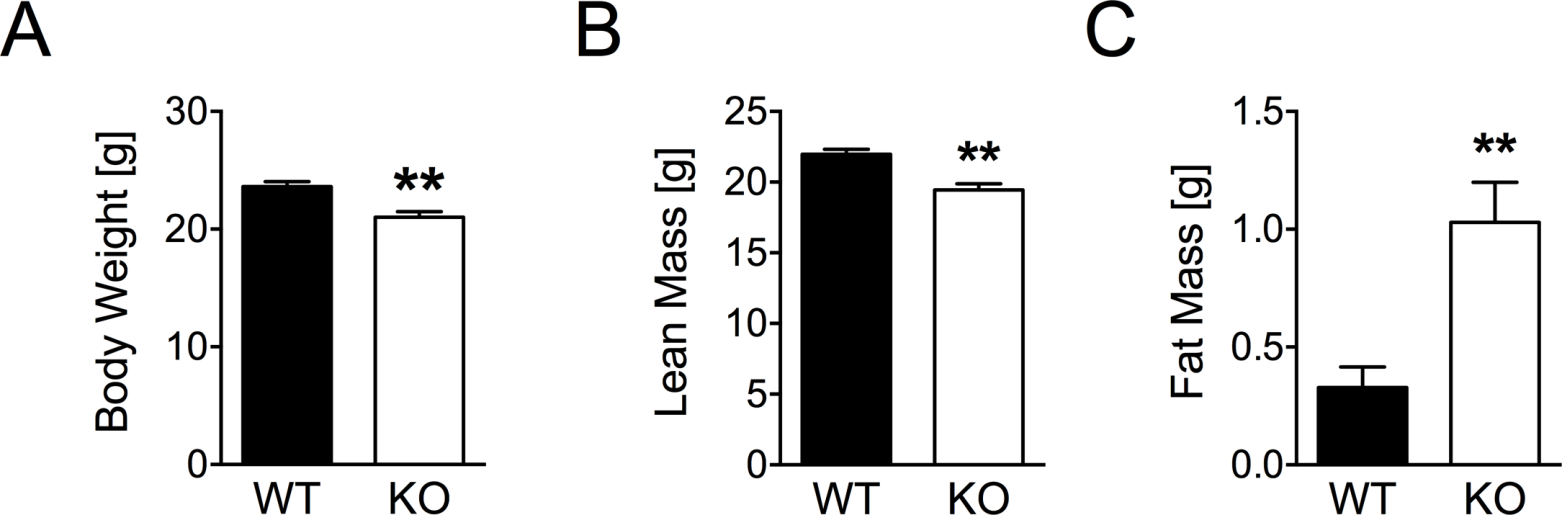
Body weight and body composition in chow-fed DUSP6 WT and KO mice. Age-matched DUSP6 WT and KO mice (Age 7.3 ± 0.3 vs. 7.6 ± 0.1 weeks) were fed standard chow-diet and monitored for their body weight (A), lean mass (B) and fat mass (C). n = 8. Means ± SEM, **p<0.01.

Young (7 weeks old) chow-fed mice with global DUSP6 deficiency of mixed C57BL/6 and 129 background were viable and fertile, but displayed significantly decreased body weight compared to chow-fed wild type mice (Fig 3A). This decrease in weight was largely attributed to decreased lean mass (Fig 3B), which may suggest early-life weight retardation. Notably, chow-fed KO mice displayed a shift in body composition towards slightly elevated fat mass (Fig 3C).

### Chronic HFD feeding normalizes lean and fat mass in Dusp6 deficient mice

Differences in body weight and body composition in chow-fed young WT and DUSP6 KO mice prompted us to assess whether DUSP6 deficiency would impair the ability of mice to cope with chronic metabolic stress due to an obesogenic dietary environment. Chow-fed young mice were placed on HFD (58% calories from fat) and monitored for changes in body weight, lean mass and fat mass.

**Fig 4:**
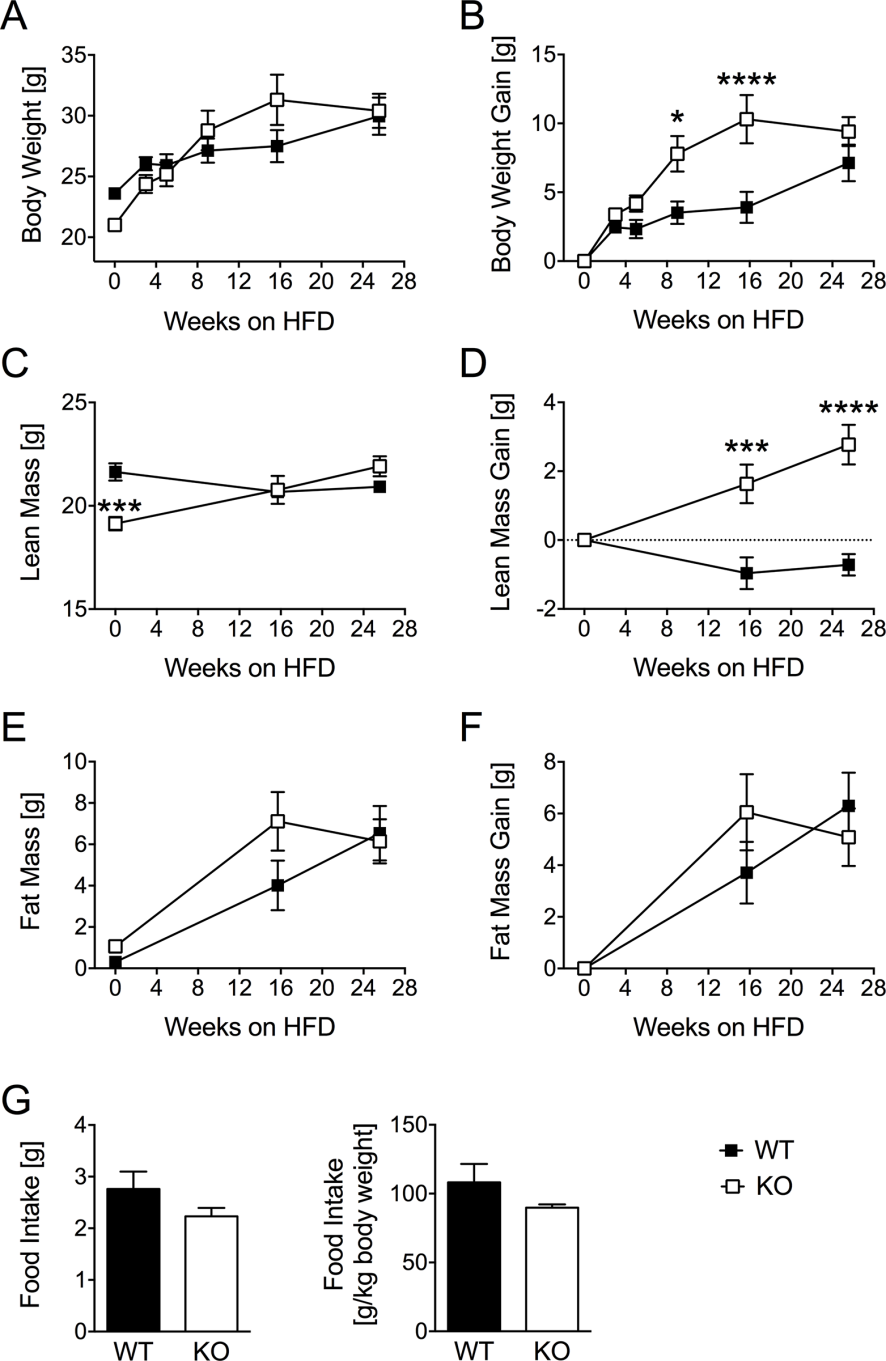
Body weight and body composition in HFD-fed DUSP6 WT and KO mice. DUSP6 WT and KO mice were exposed to high fat diet (HFD; D12331; 58% kcal fat; Research Diets Inc., New Brunswick, USA) for 25 weeks, and evaluated for changes in body weight (A,B), lean mass (C,D) and fat mass (E,F). Food intake per animal or kg body weight was monitored between weeks 3 and 5 (G). A-F: n = 8–9; G: n = 3–5 (group housed mice). Means ± SEM, ***p<0.001, ****p<0.0001.

DUSP6 KO mice placed on HFD gained significantly more body weight (Fig 4A and 4B) and lean mass (Fig 4C and 4D) compared to HFD-fed WT mice. Both genotypes gained similar amounts of fat mass during the diet exposure (Fig 4E and 4F). By the end of the 26 weeks of HFD exposure, however, we could not find any significant differences in body weight, lean mass or fat mass. Food intake in DUSP6 WT and KO mice was monitored between weeks 3 and 5 of HFD exposure, but was unchanged between genotypes when normalized per animal or per kg body weight (Fig 4G) Overall, our data suggest that Dusp6 plays a role in early stages of life and adipose tissue development. Our data further indicate that DUSP6 deficiency does not alter the susceptibility of adult mice to develop diet-induced obesity.

### Altered lipid homeostasis in DUSP6 deficient mice exposed to HFD

**Fig 5:**
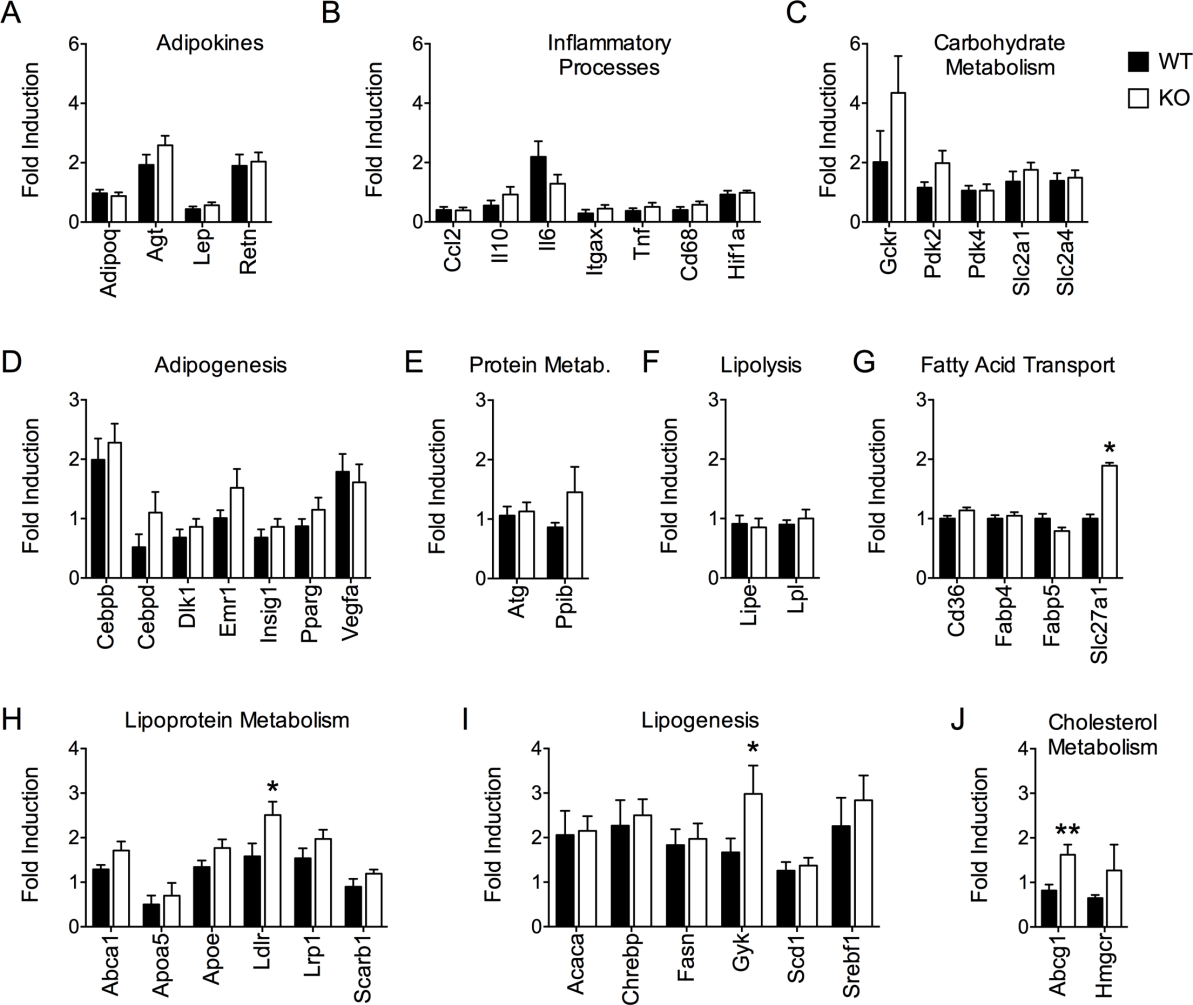
Gene profiling of epididymal white adipose tissue. Low-density microarrays for adipokines (A) and enzymes important for inflammatory processes (B), carbohydrate metabolism (C), adipogenesis (D), protein metabolism (E), lipolysis (F), fatty acid transport (G), lipoprotein metabolism (H), lipogenesis (I) and cholesterol metabolism (J) revealed a significant up-regulation of genes important for fatty acid transport (*Slc7a1*), lipogenesis (*Gyk*), lipoprotein metabolism (*Ldlr*), and steroid metabolism (*Abcg1*) in eWAT of HFD-fed Dusp6 KO mice, compared to WT controls. All results were normalized to the expression of 18S. Means ± SEM, n = 8. *p<0.05, **p<0.01.

Similar propensities for diet-induced obesity in HFD-fed WT and DUSP6 KO mice were largely reflected by similar epididymal WAT gene expression levels of key enzymes involved in adipose tissue biology and function. For instance, we observed unchanged expression levels for the adipokines adiponectin (*AdipoQ*), angiotensinogen (*Atg*), leptin (*Lep*) and resistin (*Retn*) (Fig 5A) in WT and DUSP6 KO mice after 26 weeks of HFD-exposure. Similarly, we did not detect significant differences in the expression of pro-inflammatory genes such as chemokine C-C motif ligand 2 *(Ccl2)*, interleukin 10 *(Il10)*, interleukin 6 *(Il6)*, integrin subunit alpha X *(Itgax)*, tumor necrosis factor *(Tnf)*, macrophage antigen *Cd68* or hypoxia-inducible factor 1 *(Hif1a)* between HFD-fed WT and Dusp6 KO mice (Fig 5B). Genes involved in carbohydrate metabolism (Fig 5C; glucokinase regulatory protein *(Gckr)*, pyruvate dehydrogenase kinase 2 *(Pdk2)*, pyruvate dehydrogenase kinase 4 *(Pdk4)* solute carrier family 2 member 1 and 4 *(Slc2a1*, *Slc2a4)*), adipogenesis (Fig 5D; C/EBP beta CCAAT/enhancer binding protein beta and delta *(Cebpb*, *Cebpd)*, delta-like 1 homolog *(Dlk1)*, Egf-like module containing, mucin-like hormone receptor-like 1 *(Emr1)*, insulin-induced protein 1 *(Insig1)*, peroxisome proliferator-activated receptor gamma *(Pparg)*, vascular endothelial growth factor *(Vegf)*), protein metabolism (Fig 5E; autophagy related 4 homolog C *(Atg4c)*, peptidyl-prolyl cis-trans isomerase B *(Ppib)*) or lipolysis (Fig 5F; hormone-sensitive lipase *(Lipe)*, lipoprotein lipase *(Lpl)*) remained unchanged between genotypes. HFD-fed DUSP6 KO mice displayed increased levels of fatty acid transport protein 1 (Fig 5G; *Slc27a1*), but unchanged levels of CD36 antigen *(Cd36)*, fatty acid binding protein 4 *(Fabp4)* or fatty acid binding protein 5 *(Fabp5)*, compared to HFD-fed WT controls. HFD-fed DUSP6 KO mice further displayed increased low-density lipoprotein receptor *(Ldlr)*, but other enzymes involved in lipoprotein metabolism (Fig 5H; ATP-binding cassette transporter *(Abca1)*, apolipoprotein A-V *(Apoa5)*, apolipoprotein E *(Apoe)*, low-density lipoprotein-related protein 1 *(Lrp1)* or scavenger receptor class B member 1 *(Scarb1)*) remained unchanged. The expression of lipogenesis enzymes (Fig 5I; acetyl-CoA carboxylase alpha *(Acaca)*, carbohydrate-responsive element-binding protein *(Chrebp)*, fatty acid synthase *(Fasn)*, stearoyl CoA desaturase 1 *(Scd1)*, sterol regulatory element binding protein-1 *(Srebpf1)*) remained unchanged except for glycerol kinase *(Gyk)*, which was increased in eWAT of DUSP6 KO mice. Cholesterol metabolism *(*Fig 5J) appeared to be induced in eWAT of DUSP6 KO mice, as indicated by increased ATP-binding cassette sub-family G member 1 *(Abcg1)* expression and a trend for increased 3-hydroxy-3-methyl-glutaryl-coenzyme A reductase (*Hmgcr)* levels. Overall, eWAT gene expression levels appears largely inconspicuous, which is also reflected by unchanged total ERK or phospoho-ERK protein levels (data not shown). However, a limited number of deregulated genes *(Slc27a1*, *Ldlr*, *Gyk*, *Abcg1)* points towards slightly increased cholesterol and lipid metabolism in HFD-fed DUSP6 KO mice.

### Impaired glucose homeostasis in DUSP6 deficient mice

**Fig 6:**
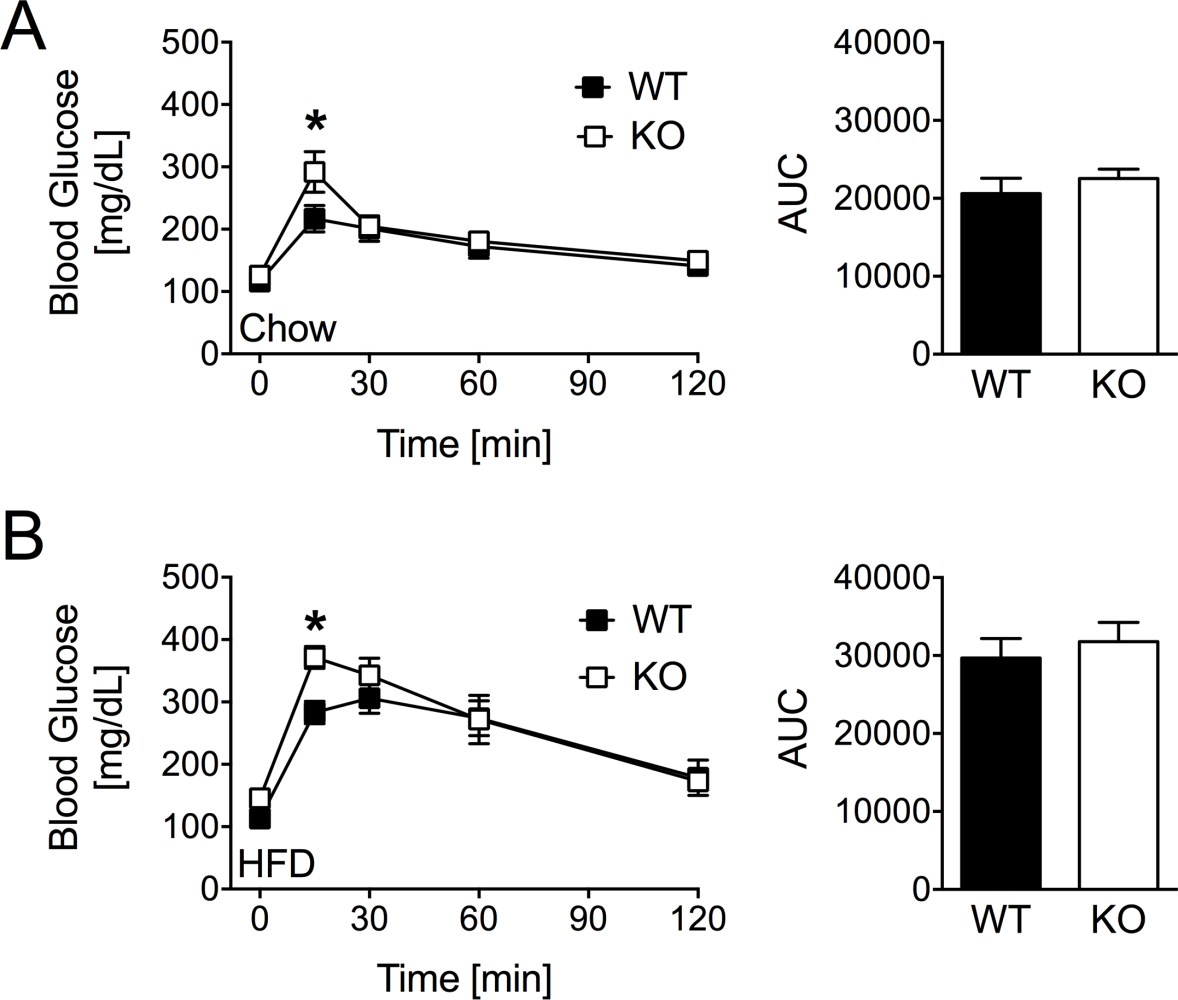
DUSP6 ablation impairs glucose tolerance. Intraperitoneal bolus injections of 2 g glucose per kg body weight led to a temporary increase in glucose excursions 15 min after injection but unchanged area under the curve levels in (A) chow-fed (n = 9–10) and (B) 16-weeks HFD-fed DUSP6 WT and KO mice (n = 9). Means ± SEM; *p<0.05.

DUSP6 was recently associated with gluconeogenic gene expression and hepatic gluconeogenesis [17], potentially mediated via direct interaction with and dephosphorylation of FOXO1 [24,25]. We here aimed to delineate the impact of germline DUSP6 ablation on systemic glucose tolerance in young and lean chow-fed DUSP6 WT and KO mice, and subsequently in WT and KO mice subjected to chronic HFD exposure. Glucose tolerance tests revealed significantly increased glucose levels 15 min after a glucose bolus in young chow-fed DUSP6 KO mice (Fig 6A, left panel). Glucose excursions quickly normalized and area under the curve (AUC) levels remained unchanged (Fig 6A, right panel). DUSP6 KO mice subjected to HFD for 16 weeks revealed the same glucose intolerance as chow-fed KO mice, i.e. unchanged baseline glucose levels, significantly increased glucose excursions 15 min after a glucose bolus, and a quick normalization of glucose levels with unchanged AUC levels (Fig 6B). Overall, these data suggest that DUSP6 deficiency impairs glucose tolerance independently of diet exposure and body weight or lean mass changes.

## Discussion

Here, we report effects of global genetic ablation of ERK-specific dual specificity phosphatase 6 on systemic energy and glucose homeostasis in mice. Our data reveals slightly increased fat mass, but reduced body weight and lean mass in young, chow-fed DUSP6 KO mice, reminiscent of early-age weight retardation. Exposure to HFD leads to a compensation of body composition in both genotypes and increased expression of genes involved in lipid and cholesterol metabolism in eWAT of DUSP6 KO mice, compared to WT controls. Regardless of body weight or body adiposity, glucose tolerance was perturbed in both chow-fed lean or HFD-fed obese DUSP6 KO, compared to their respective WT controls.

DUSP6 is undetectable in skeletal muscle and testis, but widely expressed in tissues such as lung, heart, spleen, thymus, kidney, liver, WAT and brain [19,23,28]. In the brain, Dusp6 seems to act as neuro-protectant in cells exposed to oxidative stress: primary cortical neurons with Dusp6 overexpression blocked the nuclear translocation of ERK1, leading to protection from glutamate-induced oxidative toxicity [29]. A similar protective role for Dusp6 was found in non-neuronal oligodendrocytes in the CNS, where *Dusp6* mRNA expression was increased after glutamate treatment [30]. In contrast, LPS treatment led to a reduction of *Dusp6* mRNA levels in microglia [31]. We found unaltered *Dusp6* mRNA levels in various brain areas of mice subjected to substantial metabolic challenges such as leptin injections, diet–induced obesity or prolonged fasting and refeeding. Accordingly, DUSP6, at least at the level of gene expression, does not seem to be a crucial negative feedback regulator to dampen the extent and duration of ERK signaling in response to metabolic stimuli in the selected brain areas associated with metabolic homeostasis.

Elevated fat mass in young DUSP6 KO mice points towards slightly augmented adipogenesis, potentially due to increased basal ERK activity [19] in eWAT. Notably, we were unable to find such increased ERK activation in eWAT of DUSP6 KO mice exposed to HFD for 25 weeks compared to HFD-fed WT controls. Whether deregulated ERK activity acts as driving force for earlier effects of DUSP6 deficiency on body weight gain after 9 or 16 weeks of HFD exposure remains to be tested. Overall, the long HFD exposure may facilitate compensatory mechanisms that help overcoming DUSP6 deficiency and ERK hyperactivation, e.g. via alternative phosphatases, kinases or scaffold proteins, which would explain similar body weights and body composition in WT and KO mice at the end of the monitoring period.

Unaltered *Dusp6* expression in primary preadipocytes undergoing differentiation to mature adipocytes does not support a major role of DUSP6 in adipogenesis. Rather, DUSP6 may play a role in controlling metabolism in mature adipocytes, which is in line with our finding of upregulated *Dusp6* mRNA levels in eWAT of DIO mice. Indeed, gene profiling studies in white adipose tissue show an induction of anabolic, mostly lipogenic genes in HFD-fed DUSP6 KO mice. Amongst the genes increased in eWAT of DUSP6 KO mice, we found glycerol kinase *(Gyk)*, which is absent from adipocytes under normal conditions [32]. Induction of *Gyk* expression and activity, for instance via aquaporin 7 *(Agp7)* deficiency or peroxisome proliferator-activated receptor γ *(PPARγ)* activation, results in the re-utilization of glycerol as direct source of glycerol-3-phosphatse to facilitate re-esterification of free fatty acids and a progressive accumulation of triacylglycerol towards adipocyte hypertrophy [32]. This is in line with the upregulation of low-density lipoprotein receptor *(Ldlr)* in eWAT of HFD-fed DUSP6 KO that points towards increased low-density lipoprotein uptake with elevated cholesterol, free fatty acid and lipid transport into the adipocytes. LDLR KO mice were resistant to developing diet-induced obesity when exposed to a Western diet (WD) [33,34]. However, a conflicting report suggests that LDLR KO mice are prone to high body adiposity when fed with a diabetogenic diet rich in fat and sugar [35]. Accordingly, the role of LDLR in the etiology of body adiposity remains unclear. Our hypothesis of altered lipid homeostasis in eWAT of DUSP6 KO mice is further supported by increased expression of ATP-binding cassette subfamily member 1 *(Abcg1)* that has been linked with increased cholesterol and phospholipid transport into macrophages [36]. ABCG1 was further shown to promote triglyceride storage and fat mass growth in mice, and virally mediated *Abcg1* knockdown in adipose tissue diminished adiposity and weight gain in HFD-fed mice [37]. Elevated levels of ABCG1 in eWAT of DUSP6 KOs are thus indicative of increased lipid and cholesterol flux and adiposity.

Last, we found elevated levels of solute carrier 27 a1 (*Slc27a1*), the most abundant fatty acid transporter in adipocytes, in eWAT of HFD-fed DUSP6 KO mice. SLC27A1 translocates to the plasma membrane upon insulin stimulation, where it mediates long-chain fatty acid uptake to facilitate triglyceride synthesis and lipid storage [38]. However, SLC27A1 does not appear to drive adiposity based on the similar body adiposities found in HFD-fed SLC27A1 WT and KO mice [39]. Nonetheless, since *Slc27a1* ablation was linked with higher insulin sensitivity [39], elevated *Slc27a1* expression in our HFD-fed DUSP6 KO mice suggest impaired eWAT insulin sensitivity. Indeed, we observed impairment of glucose tolerance in HFD-fed DUSP6 KO mice, but the role of eWAT in that process remains unclear. Overall, augmented lipid metabolism and an anabolic eWAT state may explain why DUSP6 KO mice displayed normalization of weight retardation after exposure to an obesogenic environment. However, more profound changes in the expression of anabolic genes may have occurred before our measurements in HFD exposure weeks 9 to 16, where KO mice show a significantly larger weight gain compared to WT mice to compensate for the early weight retardation.

In a previous report, DUSP6 KO mice exposed to HFD displayed decreased fat mass gain and lower propensity for diet-induced obesity [23]. A subsequent report corroborated the resistance to diet-induced-obesity in DUSP6 KO mice and attributed this to an alteration of the gut microbiome, which conferred obesity protection by ameliorating the gut microbiota response to diet-mediated stress [40]. Accordingly, fecal transplants of microbiota from DUSP6 KO mice significantly increased energy expenditure and reduced weight gain in recipient germ-free wild-type mice exposed to HFD [40]. Our data stand in contrast to these earlier studies, displaying similar propensities for diet-induced obesity, as evidenced by similar fat mass, lean mass and body weight in DUSP6 WT and KO mice after 26 weeks of HFD exposure. Reasons for this discrepancy remain elusive. Importantly, our study and the studies by Feng [23], Ruan [41] and colleagues were performed using mice with the same genetic mutation [19]. However, our mice had a mixed genetic background (C57Bl/6J and 129) compared to the pure C57Bl/6J background of mice used by Feng [23], Ruan [41] and colleagues (Jackson Lab strain #025564).

Both mice with a pure 129 or C57Bl/6J background were shown to be equally susceptible to DIO [41]. Others report full [42] or partial [43] protection from DIO in 129 mice compared to C57Bl/6J mice. Accordingly, a differential effect of a mixed vs. pure background on the metabolic phenotype of DUSP6 KO mice cannot be ruled out completely. Our data reveals a considerable propensity for DIO in HFD-fed WT and DUSP6 KO mice on a mixed background. Alterations in housing conditions, diets or the gut microbiological environments are known to serve as confounding factors for phenotypic disease differences [44]. For instance, differences between labs in the propensity of genetically identical Toll-like receptor KO mice to develop obesity and comorbid sequelae may be attributed to divergence of the gut microbiota after prolonged breeding in the respective housing facilities [45,46]. Our data suggests that discrepancies between reports on metabolic dysfunction in DUSP6 KO mice may underlie a similar principle. In that respect, especially the difference in the genetic background appears of major importance. Reports showed that even subtle differences in background in C57Bl/6J vs. C57Bl/6N mice can have a differential effect on obesity susceptibility, likely driven by changes in the microbiome composition [47]. Other reports highlight the genetic background as important denominator for the microbiome composition [48–50], which can have a profound impact on cardiovascular and metabolic phenotypes [42,51]. Overall, however, environmental influences appear to have a greater impact on the microbiome compared to the genetic background [49]. In fact, only 19% of the variance in murine gut microbiota composition appears to be driven by background genetics, while 31.7% are driven by the relative contribution of cage effects and 45.5% by inter-individual variations inherent to a mixed co-housing design [52].

The standardization of microbiota compositions in DUSP6 WT and KO mice between labs and mouse husbandry rooms would be the ideal solution to solve the discrepant findings. However, at current this approach does not appear feasible. Mouse husbandry facilities often implement complex barrier rules, and the colonization with a general microbiome composition will likely only be temporary until the microbiota prevalent at the respective facilities cause a drift or replacement.

Distinct gut microbiota may also explain discrepant effects of DUSP6 deletion on glucose tolerance in our study and previous reports. DUSP6 KO mice in our study developed mild glucose intolerance on chow and high fat diet. Glucoregulatory organs impaired by DUSP6 deficiency remain to be determined, but our data indicates that the effect of DUSP6 is independent from body weight or body composition. These data stand in contrast to reports in HFD-fed DUSP6 KO mice [23,40] or HFD-fed mice with an antisense oligonucleotide-driven knockdown of hepatic DUSP6 [53], which revealed improved glucose homeostasis concomitant with a protection from obesity after DUSP6 inactivation. Reasons for this discrepancy remain elusive, but may entail differential macronutrient contents in the HFDs used between reports; we used a ‘Surwit diet’ high in fat and sucrose with vegetable (coconut) oil as main source of dietary fat (58% calories from fat), while the two previous studies [23,40] used HFDs with animal fat, i.e. lard, as source of dietary fat (45% or 60% calories from fat, respectively).

Mice display a comparable reduction in microbial diversity and a shift from commensal gut microbiota towards a pro-obesogenic profile, indicated by a shift in the ratio of firmicutes to baceteroidetes, when exposed to HFD or WD (reviewed in [54]). Germ-free mice were protected from diet-induced obesity when exposed to WD which was based on high sucrose content and beef tallow as fat source [55,56]. However, when exposed to HFD with lower sucrose content and coconut oil as source for dietary fat, germ-free mice displayed a normal propensity to develop DIO compared to conventionally housed HFD-fed mice [56]. Accordingly, despite similar macronutrient content, sucrose levels and the type and source of fat in WD vs. HFD seem to determine whether alterations in the host microbiome interaction are causally linked with the development and maintenance of DIO. Accordingly, the type of HFD and potentially distinct housing conditions with differential microbiota prevalent in the animal facilities may help to explain discrepant findings between our study and previous reports in genetically identical DUSP6 KO mice.

Last, our finding of early-age weight retardation, i.e. reduced body weight and lean mass, in young DUSP6 deficient mice corroborates an earlier report showing decreased body weight in young DUSP6 KO mice [23]. Exact molecular underpinnings for the early-age weight retardation in response to DUSP6 deficiency remain unknown, but may include hyperactivation of ERK [19] at multiple growth-regulating organs, for instance at the level of the hypothalamus and pituitary. Future studies should moreover clarify the role of DUSP6 in pre- and postnatal development and in the control of lean mass and body length via the growth hormone axis.

In summary, we corroborate an earlier report showing reduced weight and lean mass in young DUSP6 KO mice. Mechanisms and sites-of-action for this early weight retardation phenotype remain elusive. We further show that DUSP6 deficiency has limited effects on the regulation of systemic energy homeostasis, but impairs glucose homeostasis in adult mice; these data stand in contrast to earlier reports which suggest protection from DIO and glucose intolerance in the absence of DUSP6, potentially mediated via alterations in the gut microbiome [23,40,53]. Importantly, all studies were conducted in mice with the same genetic mutation, but with a different genetic background of pure C57Bl/6J vs. mixed 129 x C57Bl/6J, and different types of HFD. Future studies should clarify this potential role of the genetic background, of the diet, and of host microbiome interactions for DUSP6-ERK signaling and the susceptibility for DIO and etiology of metabolic disease.

## Abbreviations

eWAT: epididymal white adipose tissue
iWAT: inguinal white adipose tissue
FFD: fat free diet
HFD: high fat diet
WD: western diet
ad lib: ad libitum
pf: pair-fed
ERK: extracellular signal-regulated kinase
DUSP6: dual specificity phosphatase 6
MAPK: mitogen activated protein kinase
MAP3K: MAP kinase kinase kinase
MAP2K: MAP kinase kinase
RAF: rapidly accelerated fibrosarcoma
MEK: MAPK/ERK: kinase
KSR: kinase suppressor of RAS
FOXO1: forkhead box protein O1
Lep^ob^ mice: leptin deficient mice
WT: wild type
KO: knockout
CNS: central nervous system
AUC: area under the curve

## References

[1] Hazzalin CA, Mahadevan LC. Mapk-regulated transcription: a continuously variable gene switch? Nat Rev Mol Cell Biol. 2002;3: 30–40. doi: 10.1038/nrm715 11823796 10.1038/nrm715

[2] Donzelli E, Lucchini C, Ballarini E, Scuteri A, Carini F, Tredici G, et al ERK1 and ERK2 are involved in recruitment and maturation of human mesenchymal stem cells induced to adipogenic differentiation. J Mol Cell Biol. 2011;3: 123–131. doi: 10.1093/jmcb/mjq050 21278199 10.1093/jmcb/mjq050

[3] Bost F, Caron L, Marchetti I, Dani C, Le Marchand-Brustel Y, Binétruy B. Retinoic acid activation of the ERK pathway is required for embryonic stem cell commitment into the adipocyte lineage. Biochem J. Portland Press Ltd; 2002;361: 621–627. 11802792 10.1042/0264-6021:3610621 PMC1222345

[4] Bost F, Aouadi M, Caron L, Even P, Belmonte N, Prot M, et al The extracellular signal-regulated kinase isoform ERK1 is specifically required for in vitro and in vivo adipogenesis. Diabetes. 2005;54: 402–411. 15677498 10.2337/diabetes.54.2.402

[5] Prusty D, Park B-H, Davis KE, Farmer SR. Activation of MEK/ERK signaling promotes adipogenesis by enhancing peroxisome proliferator-activated receptor gamma (PPARgamma) and C/EBPalpha gene expression during the differentiation of 3T3-L1 preadipocytes. J Biol Chem. 2002;277: 46226–46232. doi: 10.1074/jbc.M207776200 12270934 10.1074/jbc.M207776200

[6] de Mora JF, Porras A, Ahn N, Santos E. Mitogen-activated protein kinase activation is not necessary for, but antagonizes, 3T3-L1 adipocytic differentiation. Mol Cell Biol. American Society for Microbiology; 1997;17: 6068–6075. doi: 10.1128/MCB.17.10.6068 9315666 10.1128/mcb.17.10.6068 PMC232456

[7] Dickinson R. Diverse physiological functions for dual-specificity MAP kinase phosphatases. J Cell Sci. 2006;119(Pt 22):4607–15. doi: 10.1242/jcs.03266 17093265 10.1242/jcs.03266

[8] Costanzo-Garvey DL, Pfluger PT, Dougherty MK, Stock JL, Boehm M, Chaika O, et al KSR2 is an essential regulator of AMP kinase, energy expenditure, and insulin sensitivity. Cell Metabolism. 2009;10: 366–378. doi: 10.1016/j.cmet.2009.09.010 19883615 10.1016/j.cmet.2009.09.010 PMC2773684

[9] Kortum RL, Costanzo DL, Haferbier J, Schreiner SJ, Razidlo GL, Wu M-H, et al The molecular scaffold kinase suppressor of Ras 1 (KSR1) regulates adipogenesis. Mol Cell Biol. American Society for Microbiology; 2005;25: 7592–7604. doi: 10.1128/MCB.25.17.7592-7604.2005 16107706 10.1128/MCB.25.17.7592-7604.2005 PMC1190290

[10] Dougherty MK, Ritt DA, Zhou M, Specht SI, Monson DM, Veenstra TD, et al KSR2 is a calcineurin substrate that promotes ERK cascade activation in response to calcium signals. Mol Cell. 2009;34: 652–662. doi: 10.1016/j.molcel.2009.06.001 19560418 10.1016/j.molcel.2009.06.001 PMC2737517

[11] Casar B, Arozarena I, Sanz-Moreno V, Pinto A, Agudo-Ibáñez L, Marais R, et al Ras subcellular localization defines extracellular signal-regulated kinase 1 and 2 substrate specificity through distinct utilization of scaffold proteins. Mol Cell Biol. American Society for Microbiology; 2009;29: 1338–1353. doi: 10.1128/MCB.01359-08 19114553 10.1128/MCB.01359-08 PMC2643815

[12] Kidger AM, Keyse SM. The regulation of oncogenic Ras/ERK signalling by dual-specificity mitogen activated protein kinase phosphatases (MKPs). Semin Cell Dev Biol. 2016;50: 125–132. doi: 10.1016/j.semcdb.2016.01.009 26791049 10.1016/j.semcdb.2016.01.009 PMC5056954

[13] Patterson KI, Brummer T, O'Brien PM, Daly RJ. Dual-specificity phosphatases: critical regulators with diverse cellular targets. Biochem J. 2009;418: 475–489. 19228121 10.1042/bj20082234

[14] Jeffrey KL, Camps M, Rommel C, Mackay CR. Targeting dual-specificity phosphatases: manipulating MAP kinase signalling and immune responses. Nat Rev Drug Discov. 2007;6: 391–403. doi: 10.1038/nrd2289 17473844 10.1038/nrd2289

[15] Groom LA, Sneddon AA, Alessi DR, Dowd S, Keyse SM. Differential regulation of the MAP, SAP and RK/p38 kinases by Pyst1, a novel cytosolic dual-specificity phosphatase. EMBO J. 1996;15: 3621–3632. 8670865 PMC451978

[16] Klock A, Herrmann BG. Cloning and expression of the mouse dual-specificity mitogen-activated protein (MAP) kinase phosphatase Mkp3 during mouse embryogenesis. Mech Dev. 2002;116: 243–247. 12128234 10.1016/s0925-4773(02)00153-3

[17] Xu H, Yang Q, Shen M, Huang X, Dembski M, Gimeno R, et al Dual specificity MAPK phosphatase 3 activates PEPCK gene transcription and increases gluconeogenesis in rat hepatoma cells. J Biol Chem. American Society for Biochemistry and Molecular Biology; 2005;280: 36013–36018. doi: 10.1074/jbc.M508027200 16126724 10.1074/jbc.M508027200

[18] Liu B. Association of the dusp6 (mkp3) gene with mouse brain weight and forebrain structure. J Child Neurol. SAGE Publications; 2008;23: 624–627. doi: 10.1177/0883073807313042 18344456 10.1177/0883073807313042

[19] Maillet M, Purcell NH, Sargent MA, York AJ, Bueno OF, Molkentin JD. DUSP6 (MKP3) null mice show enhanced ERK1/2 phosphorylation at baseline and increased myocyte proliferation in the heart affecting disease susceptibility. J Biol Chem. 2008;283: 31246–31255. doi: 10.1074/jbc.M806085200 18753132 10.1074/jbc.M806085200 PMC2576531

[20] Zhai X, Han Q, Shan Z, Qu X, Guo L, Zhou Y. Dual specificity phosphatase 6 suppresses the growth and metastasis of prostate cancer cells. Mol Med Rep. Spandidos Publications; 2014;10: 3052–3058. doi: 10.3892/mmr.2014.2575 25241655 10.3892/mmr.2014.2575

[21] Messina S, Frati L, Leonetti C, Zuchegna C, Di Zazzo E, Calogero A, et al Dual-specificity phosphatase DUSP6 has tumor-promoting properties in human glioblastomas. Oncogene. 2011;30: 3813–3820. doi: 10.1038/onc.2011.99 21499306 10.1038/onc.2011.99

[22] Chan DW, Liu VWS, Tsao GSW, Yao K-M, Furukawa T, Chan KKL, et al Loss of MKP3 mediated by oxidative stress enhances tumorigenicity and chemoresistance of ovarian cancer cells. Carcinogenesis. Oxford University Press; 2008;29: 1742–1750. doi: 10.1093/carcin/bgn167 18632752 10.1093/carcin/bgn167

[23] Feng B, Jiao P, Helou Y, Li Y, He Q, Walters MS, et al Mitogen-activated protein kinase phosphatase 3 (MKP-3)-deficient mice are resistant to diet-induced obesity. Diabetes. 2014;63: 2924–2934. doi: 10.2337/db14-0066 24722245 10.2337/db14-0066 PMC4141371

[24] Wu Z, Jiao P, Huang X, Feng B, Feng Y, Yang S, et al MAPK phosphatase-3 promotes hepatic gluconeogenesis through dephosphorylation of forkhead box O1 in mice. J Clin Invest. 2010;120: 3901–3911. doi: 10.1172/JCI43250 20921625 10.1172/JCI43250 PMC2964981

[25] Jiao P, Feng B, Xu H. Mapping MKP-3/FOXO1 interaction and evaluating the effect on gluconeogenesis. PLoS ONE. Public Library of Science; 2012;7: e41168 doi: 10.1371/journal.pone.0041168 22848439 10.1371/journal.pone.0041168 PMC3405053

[26] Pfluger PT, Kabra DG, Aichler M, Schriever SC, Pfuhlmann K, García VC, et al Calcineurin Links Mitochondrial Elongation with Energy Metabolism. Cell Metabolism. 2015;22: 838–850. doi: 10.1016/j.cmet.2015.08.022 26411342 10.1016/j.cmet.2015.08.022

[27] Nogueiras R, Wiedmer P, Perez-Tilve D, Veyrat-Durebex C, Keogh JM, Sutton GM, et al The central melanocortin system directly controls peripheral lipid metabolism. J Clin Invest. 2007;117: 3475–3488. doi: 10.1172/JCI31743 17885689 10.1172/JCI31743 PMC1978426

[28] Muda M, Theodosiou A, Rodrigues N, Boschert U, Camps M, Gillieron C, et al The dual specificity phosphatases M3/6 and MKP-3 are highly selective for inactivation of distinct mitogen-activated protein kinases. J Biol Chem. 1996;271: 27205–27208. 8910287 10.1074/jbc.271.44.27205

[29] Levinthal DJ, Defranco DB. Reversible oxidation of ERK-directed protein phosphatases drives oxidative toxicity in neurons. J Biol Chem. American Society for Biochemistry and Molecular Biology; 2005;280: 5875–5883. doi: 10.1074/jbc.M410771200 15579467 10.1074/jbc.M410771200

[30] Domercq M, Alberdi E, Sánchez-Gómez MV, Ariz U, Pérez-Samartín A, Matute C. Dual-specific phosphatase-6 (Dusp6) and ERK mediate AMPA receptor-induced oligodendrocyte death. Journal of Biological Chemistry. American Society for Biochemistry and Molecular Biology; 2011;286: 11825–11836. doi: 10.1074/jbc.M110.153049 21300799 10.1074/jbc.M110.153049 PMC3064233

[31] Ham J-E, Oh E-K, Kim D-H, Choi S-H. Differential expression profiles and roles of inducible DUSPs and ERK1/2-specific constitutive DUSP6 and DUSP7 in microglia. Biochem Biophys Res Commun. 2015;467: 254–260. doi: 10.1016/j.bbrc.2015.09.180 26435497 10.1016/j.bbrc.2015.09.180

[32] Hibuse T, Maeda N, Funahashi T, Yamamoto K, Nagasawa A, Mizunoya W, et al Aquaporin 7 deficiency is associated with development of obesity through activation of adipose glycerol kinase. Proc Natl Acad Sci USA. National Acad Sciences; 2005;102: 10993–10998. doi: 10.1073/pnas.0503291102 16009937 10.1073/pnas.0503291102 PMC1182435

[33] Ngai YF, Quong WL, Glier MB, Glavas MM, Babich SL, Innis SM, et al Ldlr −/−Mice Display Decreased Susceptibility to Western-Type Diet-Induced Obesity Due to Increased Thermogenesis. Endocrinology. 2010;151: 5226–5236. doi: 10.1210/en.2010-0496 20881250 10.1210/en.2010-0496

[34] Karagiannides I, Abdou R, Tzortzopoulou A, Voshol PJ, Kypreos KE. Apolipoprotein E predisposes to obesity and related metabolic dysfunctions in mice. FEBS J. Blackwell Publishing Ltd; 2008;275: 4796–4809. doi: 10.1111/j.1742-4658.2008.06619.x 18754772 10.1111/j.1742-4658.2008.06619.x

[35] Schreyer SA, Vick C, Lystig TC, Mystkowski P, Leboeuf RC. LDL receptor but not apolipoprotein E deficiency increases diet-induced obesity and diabetes in mice. Am J Physiol Endocrinol Metab. 2002;282: E207–14. 11739102 10.1152/ajpendo.2002.282.1.E207

[36] Wei H, Tarling EJ, McMillen TS, Tang C, Leboeuf RC. ABCG1 regulates mouse adipose tissue macrophage cholesterol levels and ratio of M1 to M2 cells in obesity and caloric restriction. J Lipid Res. 2015;56: 2337–2347. doi: 10.1194/jlr.M063354 26489644 10.1194/jlr.M063354 PMC4655989

[37] Frisdal E, Le Lay S, Hooton H, Poupel L, Olivier M, Alili R, et al Adipocyte ATP-Binding Cassette G1 Promotes Triglyceride Storage, Fat Mass Growth, and Human Obesity. Diabetes. 2015;64: 840–855. doi: 10.2337/db14-0245 25249572 10.2337/db14-0245

[38] Hatch GM, Smith AJ, Xu FY, Hall AM, Bernlohr DA. FATP1 channels exogenous FA into 1,2,3-triacyl-sn-glycerol and down-regulates sphingomyelin and cholesterol metabolism in growing 293 cells. J Lipid Res. 2002;43: 1380–1389. 12235169 10.1194/jlr.m200130-jlr200

[39] Kim JK, Gimeno RE, Higashimori T, Kim H-J, Choi H, Punreddy S, et al Inactivation of fatty acid transport protein 1 prevents fat-induced insulin resistance in skeletal muscle. J Clin Invest. American Society for Clinical Investigation; 2004;113: 756–763. doi: 10.1172/JCI18917 14991074 10.1172/JCI18917 PMC351314

[40] Ruan J-W, Statt S, Huang C-T, Tsai Y-T, Kuo C-C, Chan H-L, et al Dual-specificity phosphatase 6 deficiency regulates gut microbiome and transcriptome response against diet-induced obesity in mice. Nat Microbiol. Nature Publishing Group; 2016;2: 1–12. doi: 10.1038/nmicrobiol.2016.220 27892926 10.1038/nmicrobiol.2016.220

[41] Montgomery MK, Hallahan NL, Brown SH, Liu M, Mitchell TW, Cooney GJ, et al Mouse strain-dependent variation in obesity and glucose homeostasis in response to high-fat feeding. Diabetologia. Springer-Verlag; 2013;56: 1129–1139. doi: 10.1007/s00125-013-2846-8 23423668 10.1007/s00125-013-2846-8

[42] Kreznar JH, Keller MP, Traeger LL, Rabaglia ME, Schueler KL, Stapleton DS, et al Host Genotype and Gut Microbiome Modulate Insulin Secretion and Diet-Induced Metabolic Phenotypes. Cell Reports. ElsevierCompany; 2017;18: 1739–1750. doi: 10.1016/j.celrep.2017.01.062 28199845 10.1016/j.celrep.2017.01.062 PMC5325228

[43] Mori MA, Liu M, Bezy O, Almind K, Shapiro H, Kasif S, et al A systems biology approach identifies inflammatory abnormalities between mouse strains prior to development of metabolic disease. Diabetes. American Diabetes Association; 2010;59: 2960–2971. doi: 10.2337/db10-0367 20713682 10.2337/db10-0367 PMC2963557

[44] Laukens D, Brinkman BM, Raes J, De Vos M, Vandenabeele P. Heterogeneity of the gut microbiome in mice: guidelines for optimizing experimental design. FEMS Microbiol Rev. Oxford University Press; 2016;40: 117–132. doi: 10.1093/femsre/fuv036 26323480 10.1093/femsre/fuv036 PMC4703068

[45] Ubeda C, Lipuma L, Gobourne A, Viale A, Leiner I, Equinda M, et al Familial transmission rather than defective innate immunity shapes the distinct intestinal microbiota of TLR-deficient mice. Journal of Experimental Medicine. Rockefeller University Press; 2012;209: 1445–1456. doi: 10.1084/jem.20120504 22826298 10.1084/jem.20120504 PMC3409501

[46] Vijay-Kumar M, Aitken JD, Carvalho FA, Cullender TC, Mwangi S, Srinivasan S, et al Metabolic syndrome and altered gut microbiota in mice lacking Toll-like receptor 5. Science. American Association for the Advancement of Science; 2010;328: 228–231. doi: 10.1126/science.1179721 20203013 10.1126/science.1179721 PMC4714868

[47] Walker A, Pfitzner B, Neschen S, Kahle M, Harir M, Lucio M, et al Distinct signatures of host&ndash;microbial meta-metabolome and gut microbiome in two C57BL&sol;6 strains under high-fat diet. The ISME Journal. Nature Publishing Group; 2014;8: 2380–2396. doi: 10.1038/ismej.2014.79 24906017 10.1038/ismej.2014.79 PMC4260703

[48] Ericsson AC, Davis JW, Spollen W, Bivens N, Givan S, Hagan CE, et al Effects of vendor and genetic background on the composition of the fecal microbiota of inbred mice. Heimesaat MM, editor. PLoS ONE. Public Library of Science; 2015;10: e0116704 doi: 10.1371/journal.pone.0116704 25675094 10.1371/journal.pone.0116704 PMC4326421

[49] Friswell MK, Gika H, Stratford IJ, Theodoridis G, Telfer B, Wilson ID, et al Site and strain-specific variation in gut microbiota profiles and metabolism in experimental mice. PLoS ONE. 2010;5: e8584 doi: 10.1371/journal.pone.0008584 20052418 10.1371/journal.pone.0008584 PMC2798964

[50] Kovacs A, Ben-Jacob N, Tayem H, Halperin E, Iraqi FA, Gophna U. Genotype is a stronger determinant than sex of the mouse gut microbiota. Microb Ecol. 2011;61: 423–428. doi: 10.1007/s00248-010-9787-2 21181142 10.1007/s00248-010-9787-2

[51] O'Connor A, Quizon PM, Albright JE, Lin FT, Bennett BJ. Responsiveness of cardiometabolic-related microbiota to diet is influenced by host genetics. Mamm Genome. 2014;25: 583–599. doi: 10.1007/s00335-014-9540-0 25159725 10.1007/s00335-014-9540-0 PMC4239785

[52] Hildebrand F, Nguyen TLA, Brinkman B, Yunta RG, Cauwe B, Vandenabeele P, et al Inflammation-associated enterotypes, host genotype, cage and inter-individual effects drive gut microbiota variation in common laboratory mice. Genome Biol. BioMed Central; 2013;14: R4 doi: 10.1186/gb-2013-14-1-r4 23347395 10.1186/gb-2013-14-1-r4 PMC4053703

[53] Souza Pauli LS, Ropelle ECC, de Souza CT, Cintra DE, da Silva ASR, de Almeida Rodrigues B, et al Exercise training decreases mitogen-activated protein kinase phosphatase-3 expression and suppresses hepatic gluconeogenesis in obese mice. J Physiol (Lond). 2014;592: 1325–1340. doi: 10.1113/jphysiol.2013.264002 24396063 10.1113/jphysiol.2013.264002 PMC3961090

[54] Ussar S, Fujisaka S, Kahn CR. Interactions between host genetics and gut microbiome in diabetes and metabolic syndrome. Mol Metab. 2016;5: 795–803. doi: 10.1016/j.molmet.2016.07.004 27617202 10.1016/j.molmet.2016.07.004 PMC5004229

[55] Bäckhed F, Manchester JK, Semenkovich CF, Gordon JI. Mechanisms underlying the resistance to diet-induced obesity in germ-free mice. Proc Natl Acad Sci USA. National Acad Sciences; 2007;104: 979–984. doi: 10.1073/pnas.0605374104 17210919 10.1073/pnas.0605374104 PMC1764762

[56] Fleissner CK, Huebel N, Abd El-Bary MM, Loh G, Klaus S, Blaut M. Absence of intestinal microbiota does not protect mice from diet-induced obesity. Br J Nutr. Cambridge University Press; 2010;104: 919–929. doi: 10.1017/S0007114510001303 20441670 10.1017/S0007114510001303

